# Histopathological growth pattern and vessel co-option in intrahepatic cholangiocarcinoma

**DOI:** 10.1007/s00795-024-00392-1

**Published:** 2024-07-03

**Authors:** Zihan Li, Hiep Nguyen Canh, Kenta Takahashi, Dong Le Thanh, Quynh Nguyen Thi, Rui Yang, Kaori Yoshimura, Yasunori Sato, Khuyen Nguyen Thi, Hiroki Nakata, Hiroko Ikeda, Kazuto Kozaka, Satoshi Kobayashi, Shintaro Yagi, Kenichi Harada

**Affiliations:** 1 Department of Human Pathology, Kanazawa University Graduate School of Medicine, Kanazawa, 920-8640 Japan; 2 Center of Pathology and Molecular Biology, National Cancer Hospital, Hanoi, Vietnam; 3 Department of Clinical Engineering, Faculty of Health Sciences, Komatsu University, Komatsu, Japan; 4 Department of Integrative Cancer Therapy and Urology, Kanazawa University Graduate School of Medical Science, Kanazawa, Japan; 5 Department of Diagnostic Pathology, Kanazawa University Hospital, Kanazawa, Japan; 6 Department of Radiology, Kanazawa University Hospital, Kanazawa, Japan; 7 Department of Hepato-Biliary-Pancreatic Surgery and Transplantation, Kanazawa University, Kanazawa, Japan

**Keywords:** Intrahepatic cholangiocarcinoma, Histopathological growth patterns, Vessel co-option, Immune microenvironment, Microvessel density

## Abstract

Intrahepatic cholangiocarcinoma (iCCA) exhibits different blood imaging features and prognosis depending on histology. To clarity histopathological growth patterns (HGPs) and vascularization processes of iCCA, we collected 145 surgical specimens and histologically classified them into large bile duct (LBD) (20 cases), small bile duct (SBD) (54), cholangiolocarcinoma (CLC) (35), combined SBD–CLC (cSBD–CLC) (26), and ductal plate malformation (DPM) (10) (sub)types. According to the invasive pattern at the interface between tumor and adjacent background liver, HGPs were classified into desmoplastic, pushing, and replacing HGPs. Desmoplastic HGP predominated in LBD type (55.5%), while replacing HGP was common in CLC (82.9%) and cSBD–CLC (84.6%) subtypes. Desmoplastic HGP reflected angiogenesis, while replacing HGP showed vessel co-option in addition to angiogenesis. By evaluating microvessel density (MVD) using vascular markers, ELTD1 identified vessel co-option and angiogenesis, and ELTD1-positive MVD at invasive margin in replacing HGP was significantly higher than those in desmoplastic and pushing HGPs. REDD1, an angiogenesis-related marker, demonstrated preferably higher MVD in the tumor center than in other areas. iCCA (sub)types and HGPs were closely related to vessel co-option and immune-related factors (lymphatic vessels, lymphocytes, and neutrophils). In conclusion, HGPs and vascular mechanisms characterize iCCA (sub)types and vessel co-option linked to the immune microenvironment.

**Supplementary Information:**

The online version contains supplementary material available at 10.1007/s00795-024-00392-1.

## Introduction

Intrahepatic cholangiocarcinoma (iCCA) is an aggressive biliary neoplasm, arising from the lining epithelium of the second-order intrahepatic bile ducts and bile ductules, including the peribiliary gland [1–4]. It is the second most common primary liver malignancy after hepatocellular carcinoma (HCC), accounting for about 5–15% of all primary hepatic tumors [2, 3, 5–7]. Despite its generally poor prognosis, with a low 5-year survival rate of 30%, iCCA exhibits a divergence in clinical outcomes depending on its histology [8–11]. Histologically, iCCA is further subclassified into large (bile) duct (LBD) and small (bile) duct (SBD) [2]. LBD type is speculated to originate from intrahepatic large bile ducts and often accompanies the precancerous or preinvasive neoplasms such as biliary intraepithelial neoplasia (BilIN) and intraepithelial papillary neoplasia of the bile duct (IPNB) [2, 9, 10]. In contrast, SBD type is speculated to arise from intrahepatic small bile ducts (septal and interlobular bile ducts) involving bile ductules and the canals of Hering [2, 3, 9, 12]. Moreover, the current World Health Organization (WHO) classification proposes two subtypes of SBD, cholangiolocarcinoma (CLC) and ductal plate malformation (DPM) [3, 13–16]. The SBD type generally has a better outcome survival rate than the LBD type [9, 10].

Histopathological growth patterns (HGPs) of hepatic tumors have been defined by the characteristics at the tumor periphery facing the surrounding liver parenchyma [17, 18]. In liver metastases, international consensus guidelines on HGP scoring have been established [18]. HGPs could be identified by morphology in hematoxylin and eosin (HE) stains, predicting tumor biology and the patient’s prognosis [17–20]. Although rarely discussed in iCCA, we first propose three predominant growth patterns in this study: desmoplastic, pushing, and replacing HGPs [11, 18, 21]. Liver metastases with different HGPs respond differently to anti-angiogenic therapy, potentially due to differences in blood supply mechanisms.[17, 20, 22, 23] We noticed vessel co-option as well as neo-angiogenesis as mechanisms of tumoral blood supply [11]. Neo-angiogenesis has been focused on oncology research in the past decades, linking tumor-associated angiogenesis with iCCA progression and anti-angiogenic agents for iCCA treatment [24–26]. However, anti-angiogenic therapy has not effectively targeted iCCA [27]. Vessel co-option is a non-angiogenic mode in which tumor cells hijack pre-existing blood vessels to supply their blood and nutrients, and has often been overlooked in iCCA [18, 20, 22, 23]. This blood supply mechanism was most commonly found in primary and metastatic tumors in highly vascularized organs such as the brain, lung, and liver, significantly associating with its prognosis [23, 28, 29]. Microvessel density (MVD) is often used as a histological marker to reflect tumor vasculature, involving vessel co-option as well as neo-angiogenesis [23, 28, 30]. Immunohistochemical markers associated with MVD might be useful in distinguishing vessel co-option from neo-angiogenesis. For example, CD34, a marker of vascular endothelial cells, was found to evaluate the capillarization of sinusoids and tumor neo-vascularization [28, 31, 32]. ELTD1 (epidermal growth factor, latrophilin, and seven transmembrane domain-containing protein-1) has been found to regulate angiogenesis as a member of tumor endothelial markers [32–34]. Meanwhile, regulation in the developmental and DNA damage response 1 (REDD1), induced by stress conditions such as hypoxia, is associated with increased MVD [35].

This study aimed to analyze the relationship between HGPs and the mechanisms of blood supply in iCCA histological subtypes. In addition, arterial vessel density (AVD), lymphatic vessel density (LVD), tumor-infiltrating lymphocytes (TILs), and tumor-associated neutrophils (TANs) were also evaluated from the aspect of tumor microenvironments for indicating potential roles of HGPs in iCCA progression, patient’s prognosis, and for informing the development of potential therapeutic strategies.

## Materials and methods

### Patients and tumor samples

This study is focused on patients with surgical resection for iCCA. These patients and pathological data were all from Kanazawa University Hospital in Japan, the National Cancer Hospital in Vietnam, and Hanoi Medical University Hospital in Vietnam. A total of 145 surgical resected iCCA were collected and staged according to the 8th edition of the American Joint Committee on Cancer (AJCC) staging manual [36]. Patient demographics, including age, gender, tumor size, and clinical stage, are detailed in Supplementary Tables S1 and S2. Tumor sizes were categorized into three groups: ≤ 2 cm, > 2 to ≤ 5 cm, and > 5 cm, based on reported thresholds [36, 37]. Survival data were available for 63 cases from Kanazawa University Hospital, but missing for cases from Vietnam National Cancer Hospital. iCCA measuring ≤ 2 cm was considered early-stage [37]. We excluded iCCAs with preoperative chemotherapy, radiation therapy, or with rare histological subtypes, such as adenosquamous carcinoma. Hilar cholangiocarcinoma and combined hepatocellular cholangiocarcinoma were also excluded. In addition to iCCA, 39 cases of hepatocellular carcinoma (HCC) were used as a control and their data were presented for reference. This study was approved by the Kanazawa University Human Research Committee (no.305–4), Vietnam National Cancer Hospital Research Committee (no.1533/QD-BVK), and Hanoi Medical University (no.HMUIRB1071), conforming to the 1975 Declaration of Helsinki ethical guidelines. The informed consent was waived by the committee because tumor specimens were retrospectively collected, the patient's identity was anonymous, and no further intervention was made on patients.

### Histological examination

Three pathologists (K.H, H.I, and H.C.N) classified each case into LBD type, SBD type, CLC subtype, and DPM subtype according to the current WHO classification [2]. All pathologists made the consensual decision for the final diagnosis conducted blinded to clinical outcomes. We divided iCCA cases into five groups based on the proportions of histological (sub)type components as before: LBD, pure SBD (almost 100% SBD component), combined SBD–CLC (cSBD–CLC, 5–80% CLC component), CLC (> 80% CLC component), and iCCA with DPM pattern (≥ 5% DPM component) (sub)types. According to the previous consensus, the 5% and 80% cutoffs were subjectively selected to analyze the affection of clinicopathological characteristics [11].

### Assessment of HGPs

According to international consensus guidelines, we selected and evaluated HE-stained slides containing the tumor and non-neoplastic background liver, and categorized them as desmoplastic, pushing, or replacing HGPs [18]. In cases containing multiple HGPs, the proportion of each HGP was calculated at 5% intervals by two experienced investigators (L.ZH and H.C.N). More than 50% were classified as the predominant pattern for each case according to the international consensus guideline for scoring these HGPs [18].

### Immunohistochemistry (IHC)

The slides were deparaffinized in xylene and dehydrated in ethanol. Depending on the antibodies used in this study, antigen retrieval was performed in either citrate buffer (pH 6) or Tris–EDTA buffer (pH 9) in the 95℃ microwave for 20 min. Following endogenous peroxidase blocking by methanol and 0.3% H_2_O_2_ for 15 min and non-specific binding sites blocking by the Novolink Polymer Detection Systems (Leica biosystems, Tokyo, Japan), these sections were incubated with mouse or rabbit primary antibody for specific antigens (Table 1) overnight at 4 °C and then with anti-rabbit/mouse immunoglobulins conjugated to peroxidase-labeled dextran polymer (DakoCytomation) for 1 h at room temperature. After the benzidine reaction using 0.01% H_2_O_2_ and 3,3-diaminobenzidine (DAB), sections were lightly counterstained with hematoxylin. No positive staining was obtained when the primary antibodies were replaced with an isotype-matched, nonimmunized immunoglobulin, which was used as a negative control for the staining procedures.

**Table 1:** Antibodies, sources, clones, and dilutions for immunohistochemistry

Antibody	Source	Type (clone)	Pretreatment	Dilution
ELTD1/ETL	Abcam	Mouse, monoclonal (ab219974)	MW, pH 6.0, 95 °C, 20 min	1:100
REDD1	Proteinech	Rabbit, polyclonal (10,638-I-AP)	MW, pH 6.0, 95 °C, 20 min	1:1000
CD34	Beckman Coulter, Brea, CA	Mouse, monoclonal (QBEnd10)	No	1:200
α-SMA	Dako	Mouse, monoclonal (1A4)	No	1:200
TAGLN	Abcam	Rabbit, monoclonal (ab170902)	MW, pH 6.0, 95 °C, 20 min	1:300
CD8	Dako	Mouse, monoclonal (M7103)	MW, pH 9.0, 95 °C, 20 min	1:20
CD66b	BD Biosciences	Mouse, monoclonal (555,723)	MW, pH 9.0, 95 °C, 20 min	1:300
D2-40	Dako	Mouse, monoclonal (M3619)	MW, pH 6.0, 95 °C, 20 min	1:100
Arginase 1	Nichirei	Rabbit, monoclonal (EP261)	MW, pH 9.0, 95 °C, 20 min	Pre-diluted
CK7	Dako	Mouse, monoclonal (OV-TL12/30	MW, pH 6.0, 95 °C, 20 min	1:50
CD3	Nichirei	Mouse, monoclonal (OKT-3)	MW, pH 9.0, 95 °C, 20 min	Pre-diluted

*MW* microwave; *ELTD1* epidermal growth factor, latrophilin, and seven transmembrane domain-containing protein-1; *REDD1* regulated in developmental and DNA damage response 1; *TAGLN* transgelin; *α-SMA* alpha smooth muscle actin

### Evaluation of MVD, AVD, and LVD

We evaluated MVD by using markers such as ELTD1, REDD1, and CD34, of LVD by D2-40 (podoplanin), and of AVD and unpaired AVD by α-SMA and TAGLN (Transgelin). Three vessel densities in different areas in the background liver, invasive margin, and tumor center were separately recorded. Vessel lumen structures were not required for microvessel counting, as long as the positive endothelial cells were clearly distinguished from adjacent stroma and tumor cells. Unpaired arteries were defined as those without surrounding portal veins and small bile ducts [11, 38, 39]. Utilizing Qupath (0.4.4) software, at each of the three evaluated areas, we counted their numbers in three fields with the highest densities (× 200 for MVD and LVD, × 100 for AVD and unpaired AVD) and calculated the average as their values for background liver, tumor invasive margin, and tumor center, in each slide by two independent investigators (L.ZH and H.C.N) [40]. The values were reported as the numbers of arteries or vessels per mm^2^. If the data differences exceeded 10%, they performed recalculations. The final evaluation was reached by averaging the results from the two investigators.

### Evaluation of T cells and neutrophils

T cell marker CD8 and neutrophil marker CD66b were stained. Five areas with the highest density of CD8- and CD66b-positive cells were selected in the background liver, invasive margin, and tumor center under low magnification. The number of CD8- and CD66b-positive cells per mm^2^ was calculated by averaging the values of the five fields for each area, using Qupath software (0.4.4) [40].

### Double IHC

To further visualize the connection between tumor vessels and surrounding liver vasculature, we select one tissue block of CLC by replacing HGP and cutting 300 serial sections for three-dimensional (3D) image construction. Double IHC staining was performed to identify the relationship among surrounding hepatic plates, tumor cells, sinusoids, MVD, LVD, AVD, and immune cells at the tumor invasive margin. Four sets of antibodies were utilized for the combination of Keratin 7 and CD34, Arginase-1 and ELTD1, CD3 and TAGLN, and CD66b and TAGLN (Table 1). After first immunostaining for Keratin7, Arginase-1, CD3, or CD66b using color development of diaminobenzidine (brown) as described above, the sections were incubated in citrate buffer (0.01 M, pH6) at 95 °C for 5 min to inactivate the antigenicity of the first antibody. After blocking non-specific binding sites again, each section was incubated with CD34, ELTD1, D2-40, and TAGLN antibodies, respectively, overnight at 4 °C. Then, the labeled polymeric HRP-linked antibody (DakoCytomation) was incubated at room temperature for 1 h. The Histogreen Substrate kit (green color) (Cosmo Bio Co, Japan) was used for the second color development.

### Digitizing and reconstruction processing

The double IHC-stained section was digitized using the NanoZoomer Machine (Hamamatsu Photonics KK, Hamamatsu, Japan). We selected the regions covering tumor cells and the background liver. Then, by using Qupath (https://qupath.github.io/), digital color images were converted into grayscale in the JPEG format [40]. Using Aira 6.3.0 software (Visage Imaging GmbH, Berlin, Germany), serial images of each grouped section were automatically aligned and reconstructed to 3D. The positive signal of each marker was marked with a distinct color for identification. The liver contour was automatically filled by threshold processing and then manually adjusted to realize the 3D reconstruction of the CLC sample.

### Statistical analysis

Data analysis was performed using SPSS software (version 29.0; IBM Statistics, Armonk, NY) and R software (version 4.0.5 for Mac OS X, R Foundation for Statistical Computing, 2021) programs for the univariate analysis. The relationship between MVD, LVD, AVD, and other relevant pathological parameters was compared by *χ* test, Mann–Whitney *U* test or Kruskal–Wallis test. Simultaneously, R software was used to conduct linear regression analysis to investigate the relationship between MVD and LVD with HGP percentage parameters. The Pearson correlation coefficient (r)-values denoting the correlation between groups were calculated when applicable. Kaplan–Meier survival curves and log-rank tests were used to calculate disease-free survival (DFS) and overall survival (OS). All statistical tests were two-sided, and *p* < 0.05 was deemed statistically significant, while *p* < 0.01 was considered highly significant.

## Results

### Histological characteristics and classification of iCCA

**Fig. 1:**
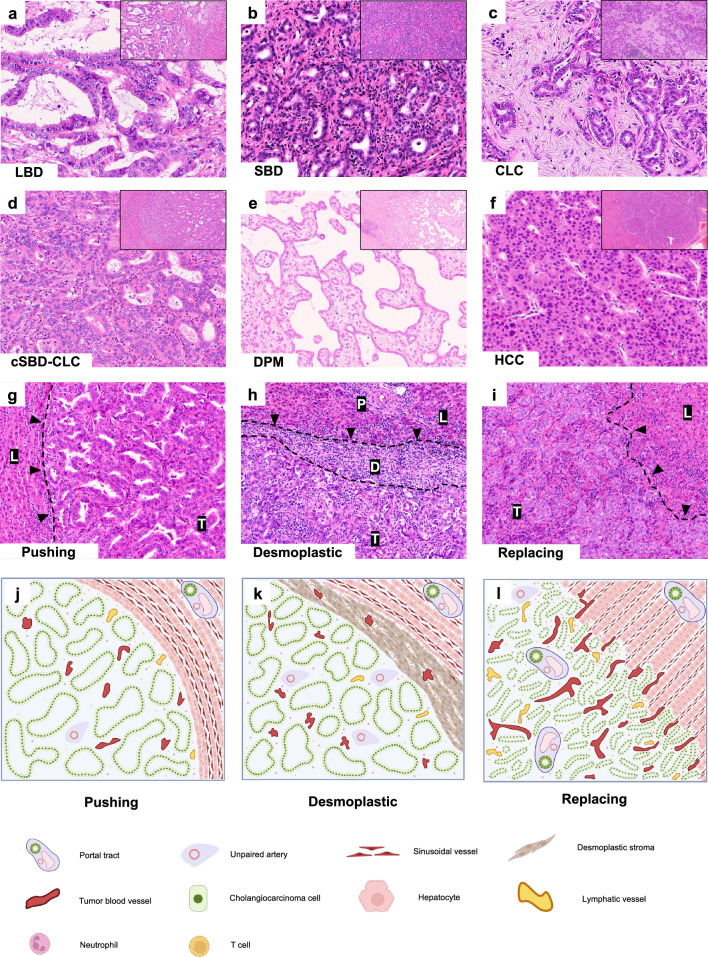
The histology classification of iCCA, with the representative images of HGPs. **a** The LBD type consists of various-sized and irregular gland structures with mucin production (HE, × 400).** b** The SBD type comprised cuboidal to low columnar tumor cells with no or minimal mucin production, showing small-sized tubular or acinar structures (HE, × 400). **c** The CLC subtype is composed of small cuboidal cells with scant cytoplasm and cord or tubular structures with “antler-like” patterns (HE, × 400). **d** The cSBD–CLC type could be observed in both SBD and CLC features in the same tumor (HE, × 400). **e** The DPM type displayed tumor epithelial cells showing cuboidal or low columnar structure, with small nucleoli and no mucin production (× 400). **f** HCC shows a thick, trabecular pattern (HE, × 400). **g** Pushing HGP is characterized by the absence of desmoplastic reaction and infiltration of liver parenchyma (dashed line), with compression and slender hepatocytes (HE, × 200). **h** Desmoplastic HGP demonstrates that the tumor (T) separates from the liver parenchyma by a desmoplastic stroma (D), with dense infiltration of immune cells (HE, × 200). The portal tract (P) is only observed in the background liver (L). **i.** Replacing HGP is characterized by tumor cells replacing hepatocytes, which take over the hepatocyte’s living space (HE, × 200). **j** The schematic representation of pushing HGP demonstrates the inconsistent arrangement of neovascularization with sinusoidal vessel.** k** The schematic representation of desmoplastic HGP shows the complete disappearance of liver structures with a neoangiogenic pattern. **i** The schematic representation of replacing HGP displays the vessel co-option pattern. *iCCA* intrahepatic cholangiocarcinoma; *HCC* hepatocellular carcinoma; *HGPs* histopathological growth patterns; *HE* hematoxylin and eosin; *LBD*, large bile duct; *SBD* small bile duct; *CLC* cholangiolocarcinoma; *cSBD-CLC* combined SBD–CLC; *DPM* ductal plate malformation; *DHGP* desmoplastic HGP; *PHGP* pushing HGP; *RHGP* replacing HGP

LBD type, presumed to originate from columnar mucous cholangiocytes, consisted of various-sized and irregular tubular structures with mucin production (Fig. 1a), and mostly showed invasive characteristics with desmoplastic stromal reactions. In contrast, the SBD type showed small-sized tubular or acinar structures (Fig. 1b), comprised of cuboidal to low columnar tumor cells with no or minimal mucin production, and relatively mild cellular atypia, compared with those of the LBD type (Fig. 1b). CLC subtype is similar to the canals of Hering in morphology and comprised small cuboidal cells with scant cytoplasm (Fig. 1c). As the characteristic proliferative pattern, cord or tubular structures with “antler-like” patterns were found. In the cSBD–CLC cases (Fig. 1d), the CLC component predominantly appeared at the tumor periphery, while the SBD component was preferably found at the tumor center. DPM is characterized by a unique biliary structure mimicking that found in Caroli’s disease or congenital hepatic fibrosis and consist of cuboidal or low columnar epithelium with small nucleoli and no mucin production. The DPM subtype had irregular lumens of varying dilation and shapes, occasionally with microcystic dilation and various degrees of dense fibrotic stroma (Fig. 1e) [16]. According to these morphological features, we assessed the presence and proportion of subtypes in each sample of iCCA. As shown in Supplemental Table 1, the case numbers of LBD, SBD, cSBD-CLC, CLC, and DPM (sub)types were 20, 54, 26, 35, and 10, respectively.

### Description and frequency of HGPs in iCCA

Pushing HGP was a growth pattern in which tumor cells compressed and pushed the adjacent hepatic column without a rim of desmoplastic pseudo-capsule (Fig. 1g and j). The liver plates surrounding tumors and the adjacent hepatocytes directly contacted tumor cells and were compressed. Predominant pushing HGP was observed in 3/20 cases (15.0%) of the LBD type and 7/54 (13.0%) of the SBD type, but only in 2/26 (7.7%) of the cSBD–CLC subtype, 2/35 (5.7%) of the CLC subtype, and 1/10 (10%) of the DPM subtype (Supplemental Table 1).

Desmoplastic HGP was characterized by a desmoplastic matrix surrounding the tumoral nodule, separating tumor cells from the surrounding hepatic parenchyma (Fig. 1h and k). No direct contacts between tumor cells and hepatocytes were found. A dense infiltration of various typed immune cells was present at the surrounding desmoplastic rims. The original hepatic lobular architecture completely disappeared within tumor nodules. Likewise, the non-neoplastic proliferation of bile ductules “ductular reaction” was sometimes observed in the desmoplastic rim. This desmoplastic HGP was present in 11/20 cases (55.0%) of LBD type (Supplemental Table 1), of which two showed pure desmoplastic HGP. The numbers of predominant desmoplastic HGP cases were relatively lower or rare in other iCCA (sub)types: 16/54 (29.6%) of the SBD type, 2/26 (7.7%) of the cSBD–CLC subtype, 4/35 (11.4%) of the CLC subtype, and 2/10 (20.0%) of the DPM subtype (Supplemental Table 1).

Replacing HGP was defined by the direct invasion of the tumor into normal hepatic parenchyma along the original hepatic column and hepatocytes were replaced by tumor cells (Fig. 1i and l), indicating replacing HGP co-opts sinusoids for vascularization and obtaining blood supply. Moreover, the portal tracts were entirely retained within tumor nodules, and overall original hepatic lobular structures were preserved. This HGP pattern was preferably found in 7/10 cases (70.0%) of the DPM subtype, 29/35 (82.9%) of the CLC subtype, 22/26 (84.6%) of the cSBD–CLC subtype, and 31/54 (57.4%) of the SBD type and, to a lesser extent, in 6/20 cases (30%) of the LBD type (Supplemental Table 1).

### Correlation between the MVD and iCCA subtypes

We speculate that tumor-related neo-vascularization might differ among iCCA (sub)types and HGPs. To clarify this objective difference, we evaluated MVD using three vascular markers, ELTD1, CD34, and REDD1.

**Fig. 2:**
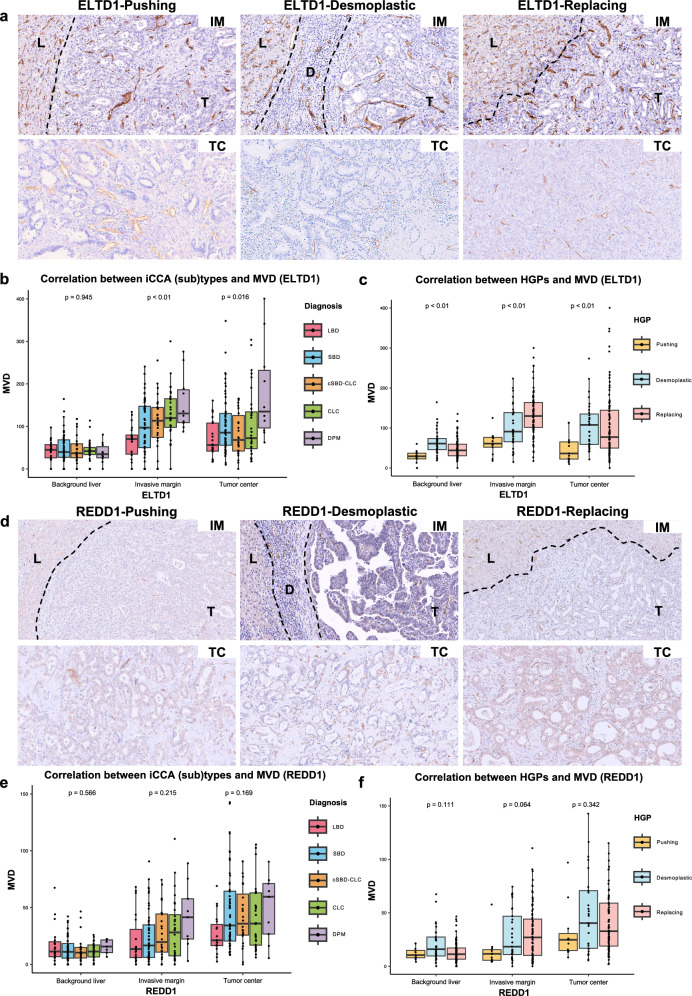
Immunohistochemical staining of ELTD1 and REDD1, and the correlation between MVD with iCCA (sub)types and HGPs types. **a** The representative images of IHC staining for invasive margin and tumor center by ELTD1. ELTD1 was strongly positive on tumor vessel endothelial and the sinusoid epithelial cells (× 200). **b** Correlation analysis between iCCA (sub)types with MVD by ELTD1. **c** Correlation analysis between HGPs with MVD by ELTD1. **d** The representative images of IHC staining for invasive margin and tumor center by REDD1 (× 200). **e** Correlation analysis between iCCA (sub)types and MVD by REDD1. **f** Correlation analysis between HGPs and MVD by REDD1. *IHC* immunohistochemical staining; *iCCA* intrahepatic cholangiocarcinoma; *HGPs* histopathological growth patterns; *MVD* microvessel density; *IM* invasive margin; *TC* tumor center; *L* background liver, *T* tumor; *D* desmoplastic stroma. Kruskal–Wallis test was used to compare groups

ELTD1 was expressed in vascular endothelial cells of both tumoral blood vessels and sinusoidal endothelial cells in the background liver, although the expression was notably enhanced in tumoral vessels compared to sinusoidal cells of the background liver (Fig. 2a and Supplemental Fig. 1a). As shown in Fig. 2b, the numbers of ELTD1-positive MVD were statistically significantly increased at both invasive margin and tumor center in all iCCA (sub)types, with the lowest density in the LBD type and the highest density in the DPM subtype.

CD34 was prominently expressed in tumoral vessels (Supplemental Fig. 1b). As summarized in Supplemental Table 1, the results of MVD evaluated by CD34 displayed the same trend as those by ELTD1. To simplify the explanation and enhance clarity, the results evaluated by CD34 were omitted in the “Results”.

REDD1 expression, reportedly related to the hypoxia condition, was found in the hepatic arteries of portal tracts and tumoral blood vessels, but not co-opted sinusoid-like vessels (Fig. 2d) [35]. Tumor REDD1-positive MVD in the DPM subtype, at both the invasive margin and tumor center, was the highest among the iCCA (sub)types and the figures were also higher than those in the background liver (Fig. 2e).

### Correlation between MVD and HGPs

To examine the potential differences in the mechanisms of tumor blood supply in iCCA, we correlated between MVD and HGPs. As shown in Fig. 2b, c, e, and f, and Supplemental Table 2, the mean numbers of MVD at the invasive margin were gradually increased in the order of pushing, desmoplastic, and replacing HGPs, irrespective of the evaluated vascular markers (ELTD1 and REDD1), with the highest value in replacing HGPs (*p* < 0.01). In replacing HGP, moreover, ELTD1-positive MVDs at the invasive margin exceeded those at the tumor center (Fig. 2b and Supplemental Table 2). On morphology, ELTD1-positive tumor vessels were directly connected to the surrounding hepatic sinusoids, while the tumor cells were directly connected to pre-existing hepatic plates at the replacing HGP invasive margin (Fig. 2a), indicating that the original hepatic sinusoidal vessels were retained or co-opted in tumors with replacing HGP.

REDD1-positive MVDs were increased at both the invasive margin and tumor center compared to background liver, yet consistently lower than ELTD1-positive MVDs irrespective of HGPs (Fig. 2c and f, and Supplemental Table 2). REDD1-positive MVDs at the invasive margin and tumor center in replacing HPG were comparable. Morphologically, REDD1-positive vessels did not appear to be co-opted sinusoids, but newly formed tumoral vessels. Therefore, in replacing HGP, the blood supply is possibly obtained through vessel co-option and neo-angiogenic vasculature, with the predominance of vessel co-option at the invasive margin. In desmoplastic HGP, ELTD1- and REDD1-positive MVDs were increased at both the invasive margin and tumor center, compared to those at the background liver, with REDD1-positive MVD higher at the tumor center than at the invasive margin (Fig. 2c and f, and Supplemental Table 2). These findings suggested that, in desmoplastic HGP, the tumor center was under more hypoxic conditions, inducing REDD1-associated angiogenesis. The MVD in pushing HGP was constantly lower than those in desmoplastic and replacing HGPs, with almost similar levels of background liver, irrespective of the usage of any vascular markers (ELTD1 and REDD1), suggesting a hypovascular tumor (Fig. 2c and f, and Supplemental Table 2).

### Correlation between total and unpaired AVD and iCCA subtypes

**Fig. 3:**
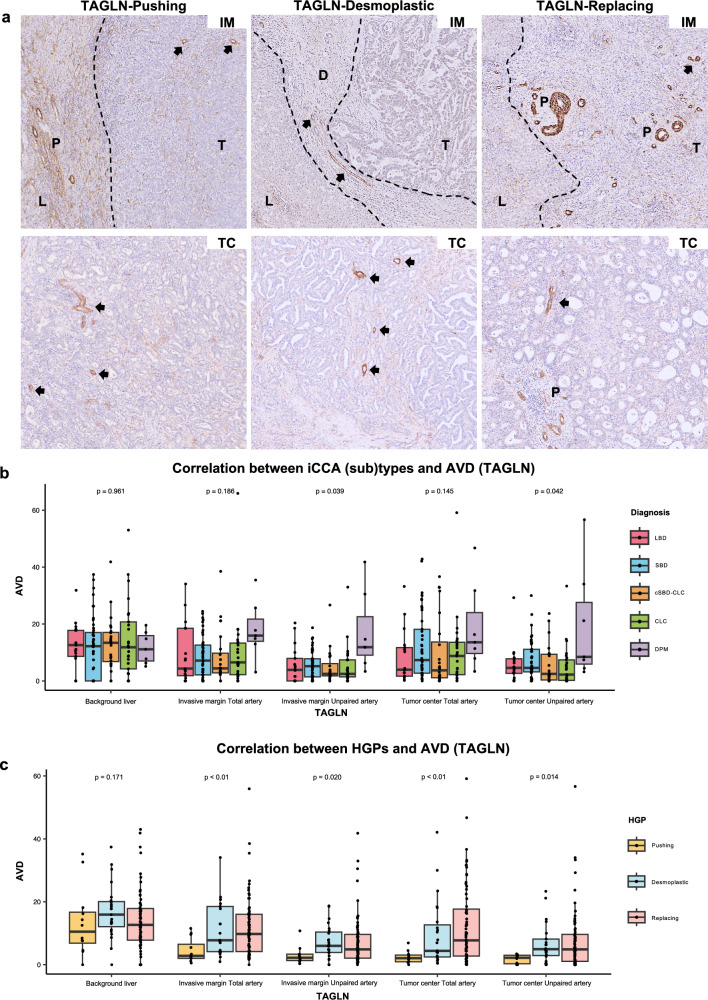
Immunohistochemical staining of TAGLN, and the correlation between AVD with iCCA (sub)types and HGPs types. **a** The representative images of IHC staining for invasive margin and tumor center by TAGLN. Within the invasive margin and tumor center, unpaired arteries (arrows) and paired arteries (P) were present (× 100). **b** Correlation analysis between iCCA (sub)types and AVD by TAGLN. **c** Correlation analysis between HGPs and AVD by TAGLN. *IHC* immunohistochemical staining; *iCCA* intrahepatic cholangiocarcinoma; *HGPs* histopathological growth patterns; *AVD* arterial vessel density; *IM* invasive margin; *TC* tumor center; *L* background liver; *T* tumor; *D* desmoplastic stroma; *P* portal tract. Kruskal–Wallis test was used for comparison between groups

In the background liver, hepatic arteries are located in portal tracts, accompanied by bile duct and/or portal veins, known as paired arteries. Unpaired arteries (abnormal vessels) appear in various hepatic tumors, reflecting tumor vascularization as a histological hallmark of angiogenesis. As shown in Fig. 3a and Supplemental Fig. 1d and e, both α-SMA and TAGLN could effectively highlight arteries and smooth muscular layers of larger veins, including unpaired arteries. However, α-SMA was also expressed in stromal components, and its AVD values were constantly higher than those obtained using TAGLN (Supplemental Table 1 and 2). Therefore, AVD data using TAGLN are shown. The DPM subtype had the highest arterial densities among iCCA (sub)types in both paired (vessel co-option) and unpaired (angiogenesis) arteries (Fig. 3b and Supplemental Table 1). The differences in total AVD at both the invasive margin and tumor center, however, were not statistically significant among iCCA (sub)types.

### Correlation between total and unpaired AVD and HGPs

As shown in Fig. 3a, c and Supplemental Table 2, TAGLN-positive AVD at the invasive margin and tumor centers was increased progressively from pushing, to desmoplastic, and to replacing HGPs, but there was no significant difference in the number of unpaired AVD between the desmoplastic and replacing HGPs. Compared to desmoplastic HGP, the increase in total AVD in replacing HGP predominantly relied on paired AVD, suggesting a significant role of vessel co-option. The ratios of unpaired AVD/paired AVD at the tumor center are 2.2/1.5, 4.9/3.9, and 4.9/6.3 for pushing, desmoplastic, and replacing HGPs, respectively (Fig. 3a and c, and Supplemental Table 2). Conversely, in pushing HGP, total and unpaired AVD values were at constantly low levels.

### *Relationship between HGPs and iCCA (sub)types versus tumor-associated lymphatic vessels, CD8* + *T cells, and neutrophils*

**Fig. 4:**
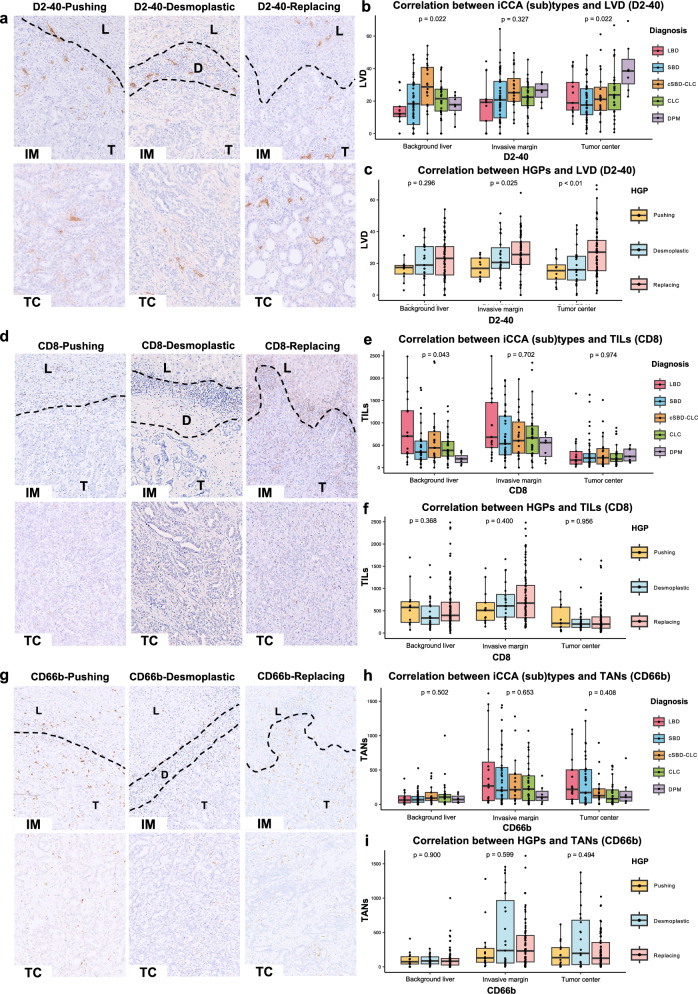
Immunohistochemical staining of D2-40, CD8, and CD66b, and the correlation between LVD and immune density with iCCA (sub)types and HGPs types. **a** The representative images of IHC staining for invasive margin and tumor center by D2-40 (× 200). **b** Correlation analysis between iCCA (sub)types and LVD by D2-40. **c** Correlation analysis between HGPs and LVD by D2-40. **d** The representative images of IHC staining for invasive margin and tumor center by CD8 (× 200). **e** Correlation analysis between iCCA (sub)types and TILs density by CD8. **f** Correlation analysis between HGPs types and TILs density by CD8. **g** The representative images of IHC staining for invasive margin and tumor center by CD66b (× 200). **h** Correlation analysis between iCCA types and TANs density by CD66b. **i** Correlation analysis between HGPs and TANs density by CD66b. *IHC* immunohistochemical staining; *iCCA* intrahepatic cholangiocarcinoma; *HGPs* histopathological growth patterns; *LVD* lymphatic vessel density; *IM* invasive margin; *TC* tumor center; *TILs* tumor-infiltrating lymphocytes; *TANs* tumor-associated neutrophils; *L* background liver; *T* tumor; *D* desmoplastic stroma. Kruskal–Wallis test was used for comparison between groups

By evaluating D2-40-positive lymphatic endothelial cells, LVD in the DPM subtype was significantly increased at the tumor center (Fig. 4a, b, and Supplemental Table 1) compared to those of other histological subtypes. According to HGPs, LVDs in replacing HGP were constantly higher at the invasive margin and tumor center, compared with those of desmoplastic and pushing HGPs (Fig. 4a and c, and Supplemental Table 2). These results suggest that the DPM subtype and replacing HGP exhibited higher LVDs, indicating the participation with more immunological properties.

To clarify if tumor-infiltrating immune cells are associated with angiogenesis, we evaluated CD8 + cytotoxic T cells and CD66b + neutrophils using immunohistochemistry. In all iCCA histological (sub)type, the CD8 + cells were higher at the invasive margin, particularly in the DPM subtype, compared to those at the tumor center and background liver, but the differences were not significant among histological (sub)types (Fig. 4d and e and Supplemental Table). The density of CD8 + cells at the invasive margin tended to be higher in iCCA with prominent replacing HGPs than those with pushing or desmoplastic HGPs, but the trend did not reach statistical significance (Fig. 4d and f and Supplemental Table 2). Although there were no statistically significant differences in CD66b + neutrophil counts among iCCA (sub)types and HGPs, the figures were relatively low at both the invasive margin and tumor center in the DPM subtype, compared to those in LBD and SBD. Further, CD66b + neutrophil densities were significantly higher in the tumor parenchyma than in the background liver, irrespective of the histological (sub)types or HGPs (Fig. 4g, h, and i and Supplemental Tables 1 and 2).

### Association between HGPs versus iCCA (sub)types and vasculature

**Fig. 5:**
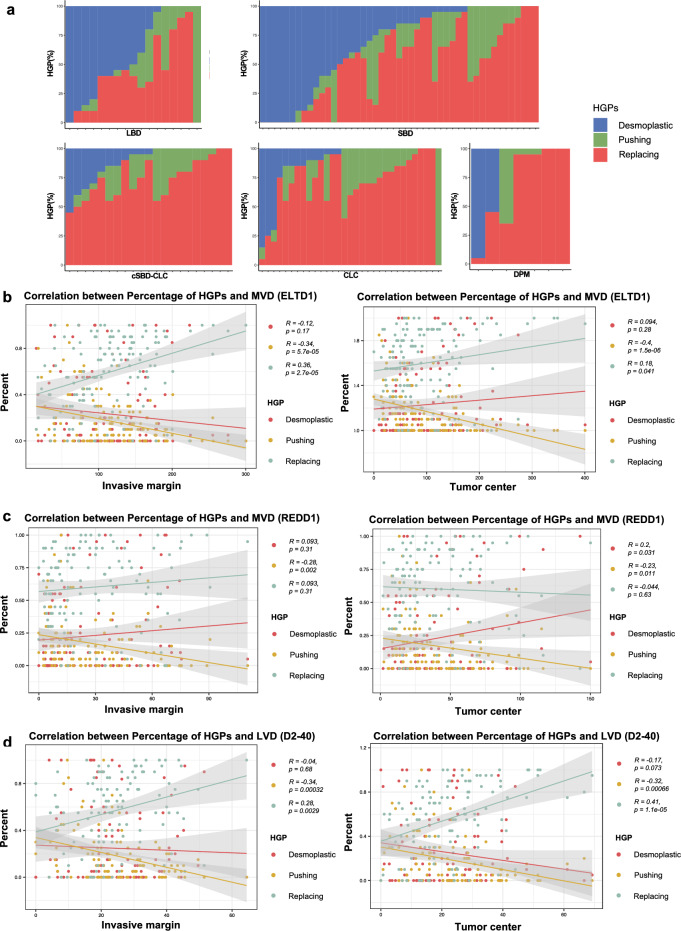
Percentage of HGPs and correlation with iCCA subtypes, MVD and LVD. **a** The relationship between the proportion of HGPs of iCCA types. The proportion (%) of each HGP is shown on the Y-axis. Each column represents a patient: desmoplastic HGP in blue, pushing HGP in green, and replacing HGP in red. **b** Scatter plot of MVD in ELTD1 and the percentage of HGPs, with the linear regression analysis. **c** Scatter plot of MVD in REDD1 and the percentage of HGPs, with the Linear Regression analysis. **d** Scatter plot of LVD in D2-40 and the percentage of HGPs, with the Linear Regression analysis. *HGPs* histopathological growth patterns; *iCCA* intrahepatic cholangiocarcinoma; *MVD* microvessel density; *LVD* lymphatic vessel density; *R* regression coefficient

As some tumors exhibit more than one HGP, we evaluated the proportions of the three HGPs in each iCCA with correlation to histological (sub)types and vasculature. As shown in Fig. 5a, replacing HGP was predominantly observed across iCCA (sub)types, particularly in the cSBD–CLC, CLC, and DPM subtypes, but not in the LBD types. As for the vasculature, at the invasive margin, ELTD1-positive vessels significantly increased with the increased proportion of replacing HGP (*R* = 0.36, *p* < 0.01), while REDD1-positive vessels displayed only a slight upward trend (*R* = 0.093, *p* = 0.31) (Fig. 5b and c). Moreover, at the tumor center, ELTD1-positive vessels slightly increased with the increased proportion of replacing HGP (*R* = 0.18, *p* < 0.05), but REDD1-positive vessels remained unchanged (*R* = − 0.044, *p* = 0.63) (Fig. 5b and c). These results support that the increased MVD in replacing HGP is mainly caused by vessel co-option. In contrast, with an increased proportion of desmoplastic HGP, ELTD1-positive vessels decreased (*R* = − 0.12, *p* = 0.17). REDD1-positive vessels slightly increased (*R* = 0.093, *p* = 0.31), respectively, at the invasive margin, with slight (*R* = 0.094, *p* = 0.28) and significant increase (*R* = 0.2, *p* < 0.05) at the tumor center (Figs. 5b and c). These results support that the increased MVD in desmoplastic HGP is mainly caused by angiogenesis. Because REDD1 stimulates angiogenesis in hypoxic environments, increased REDD1-positive vessels might reflect hypoxia in desmoplastic HGP, particularly at the tumor center. Finally, with the increased proportion of pushing HGP, ELTD1- and REDD1-positive vessels constantly decreased (Fig. 5b, c).

For LVD, the number of D2-40-positive lymphatic vessels were significantly increased with the increase of the proportion of replacing HGP increased, irrespective of the invasive margin and tumor center, but the opposite trends were found for desmoplastic and pushing HGP (Fig. 5d).

### Associations between histological (sub)types, HGPs, MVD/LVD, and other clinicopathological features

iCCA (sub)types and HGPs present distinct clinicopathological profiles. The DPM subtype was more likely to be associated with early-stage tumors (≤ 2 cm), whereas larger tumors more frequently correlated with the cSBD–CLC and SBD (sub)types (Supplemental Table 1). iCCA with pushing HGP had a significantly larger size than desmoplastic or replacing HGP tumors, with none under 2 cm observed (Supplemental Table 2). LBD subtype primarily appeared in stage 3, while there were no significant differences in staging of other (sub)types (*p* = 0.398) (Supplemental Table 1). At the same time, pushing HGP tumors were often classified into stage 3 or above (76.9%). Desmoplastic iCCAs are more prevalent in tumors under 2 cm (15.4%) compared to replacing HGP (9.4%, *p* < 0.05) (Supplemental Table 2). Although not statistically significant, the proportion of replacing and desmoplastic components tended to inversely correlate with tumor size, with the trend of replacing (*R* = − 0.16, p = 0.073) being more obvious than that of desmoplastic (*R* = − 0.11, *p* = 0.23) (Supplemental Fig. 2a). Increased replacing HGP proportions tended to be associated with lower AJCC stages, better disease-free survival (DFS) time, and enhanced overall survival (OS) time than desmoplastic HGP and pushing HGP, despite our limitation in survival data (Supplemental Fig. 2b, f, and h). Increased CLC proportion generally correlated to a better OS and disease-free survival time (Supplemental Fig. 2e and g). Conversely, an increased pushing component aligns with larger tumor sizes (p < 0.01) and advanced stages (p < 0.05) (Supplemental Figs. 2a and b). Notably, whether at the invasive margin or tumor center, increased tumor size consistently reduced MVD and LVD.

### Double IHC and 3D imaging analysis of vessel co-option in iCCA

**Fig. 6:**
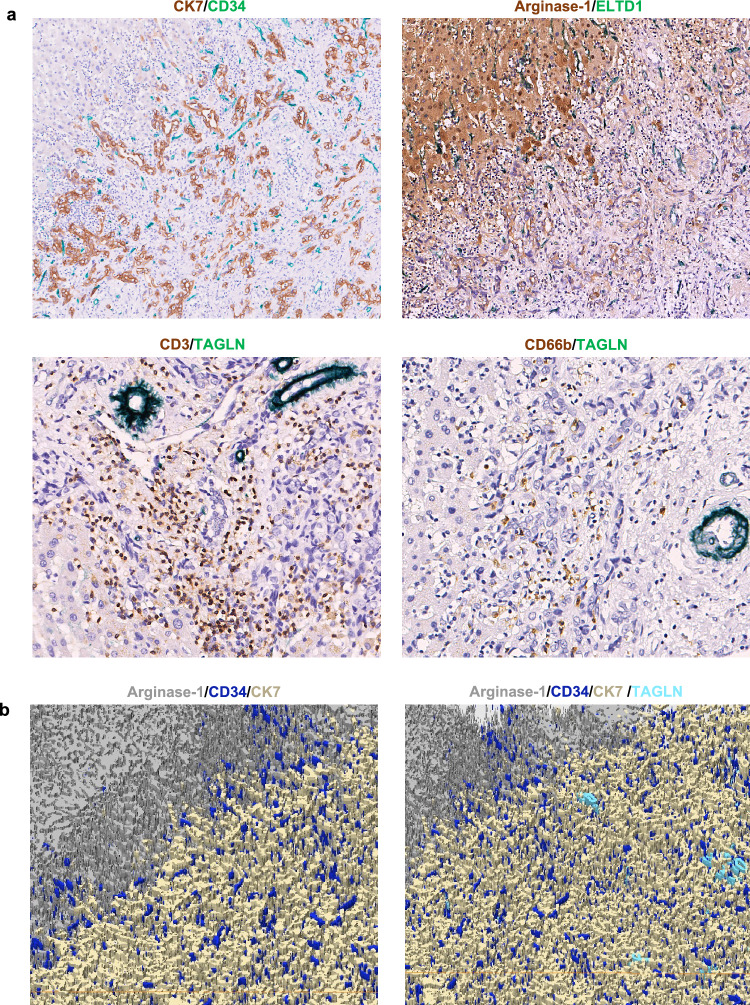
Double IHC staining of 100% replacing HGP–CLC samples as well as 3D visualization models.** a** The pure replacing HGP–CLC sample was serially sectioned and divided into four groups for double IHC staining. Double IHC of Keratin 7 (CK7) and CD34: CD34-positive co-opted vessels (green) are seen around Keratin 7-positive cholangiocarcinoma (brown). Double IHC of Arginase-1 and ELTD1: ELTD-1 vessel co-option (green) in tumor and also ELTD1-positive sinusoidal endothelial cells (capillization, green) in Arginase-1-positive hepatocytes (brown) are seen. Double IHC of CD3 and TAGLN: CD3-positive T cells (green) accumulated brown TAGLN-positive paired arteries (green) in the invasive margin. Double IHC of CD66b and TAGLN: CD66b-positive neutrophils (brown) are scattered, avoiding the area around TAGLN-positive arteries (green). **b** 3D image demonstrated that, at the invasive margin in replacing HGP, tumor cells (CK7) were closely opposed to the tumoral microvessel (TAGLN) and hepatic sinusoids and co-opted vessels (CD34) and absorb portal tracts within the tumor

To gain further insight into the mechanism of vessel co-options in tumor development, we performed double IHC staining on 300 serial sections of a CLC exhibiting entirely replacing HGP. Double IHC staining for Keratin 7 and CD34 revealed that Keratin 7-positive tumor cells were accompanied by CD34-positive hepatic sinusoid-like vessels, mimicking the original structure of the hepatic plate (Fig. 6a). Moreover, double IHC staining for Arginase-1 and ELTD1 showed that ELTD1-positive sinusoid-like vessels extended from Arginases-1-positive surrounding liver tissue into the tumor, allowing tumor cells to utilize vessel co-option to supply blood (Fig. 6a). Double IHC staining for CD3 and TAGLN indicated that CD3-positive T cells accumulated at the invasive margin, where TAGLN-positive paired and unpaired arteries were presented (Fig. 6a). Furthermore, double IHC staining for CD66b and TAGLN revealed that in invasive margins of replacing HGP, CD66b-positive neutrophils scattered without TAGLN-positive arteries (Fig. 6a). Subsequently, we performed 3D image construction to highlight the structure of vessel co-option. Consequently, these images demonstrated that at the invasive margin in replacing HGP, Keratin 7-positive cholangiocarcinoma cells were closely opposed to CD34-positive sinusoid-like vessels (vessel co-option) in addition to TAGLN-positive tumoral vessels (Fig. 6b).

## Discussion

The prognosis of iCCA patients is generally poor, and many patients suffer from recurrence even after treatment [8, 41]. However, previous studies showed that the prognosis for iCCA varies by histological (sub)type, with the SBD type typically having a more favorable outcome compared to the LBD type [9, 10, 42]. Our findings demonstrate that SBD and its subtypes are generally present with a more favorable AJCC stage, and OS and DFS time compared to the LBD type. Interestingly, the CLC and DPM subtypes of SBD generally have a better prognosis than pure SBD type [11, 43–45]. The CLC subtype was relatively rare (19.0%) and often had an overlap in histological features of the SBD type [3, 11, 46]. DPM subtype was rarer in this study (Supplemental Table 1), just 5.4%, and its tumor cells are benign looking, which histopathologically may indicate a good prognosis [45]. It also constituted a higher proportion of early-stage iCCA cases than other (sub)types (30.0%). Meanwhile, iCCA is characterized by its different histological components. This study identified that the most common mixed type was the cSBD–CLC subtype, occurring in 14.1% of iCCA cases (Supplemental Table 1). In this subtype, even if the proportion of CLC is small, the cSBD–CLC subtype will have better OS and DFS than pure SBD and pure LBD types, with increased CLC components associated with longer OS [3, 11, 43].

In this study, we aimed to clarify the relationship between the (sub)types of iCCA, tumor vasculature, and HGPs, which are recognized as predictive biomarkers and associated with different blood supply patterns [17–21, 47]. Most cases of DPM, CLC, and cSBD–CLC subtypes adopted the replacing HGP (Fig. 5a). In contrast, desmoplastic HGP was more preferably found in LBD and pure SBD types, while predominant pushing HGP was rare (Supplemental Table 1). In liver metastases, desmoplastic or pushing HGP tumors adopt mainly angiogenic blood supply. In contrast, replacing HGP tumors are vascularized mainly via vessel co-option and demonstrate typically poor response to anti-angiogenic therapy [17, 18, 20]. However, our data reveal that replacing HGP iCCAs had smaller tumor sizes and lower AJCC stages (Supplemental Table 2). An increase in tumor size was negatively correlated with the proportion of replacing HGP. Further, despite limitations in our survival data, replacing HGP iCCAs show better prognostic trends compared to those with desmoplastic and pushing HGPs. However, the impact of HGPs on outcomes of iCCA requires further investigation. To clarify this discrepancy, we speculated that the difference in HGPs might be related to differences mechanisms of blood supply and immune response, reflecting variations in OS among iCCA (sub)types.

Similarly, here we show in iCCAs that tumor cells in replacing HGP replaced the pre-existing hepatocytes in the hepatic column, but retained sinusoid-like vessels around the tumor. Double immunohistochemistry and 3D image analysis further clarified that, in replacing HGP-typed iCCA, tumor cells replaced surrounding normal hepatocytes within the hepatic column and the co-opted hepatic sinusoids extended into the tumor (Fig. 6). Moreover, in replacing HGP, portal tracts were often embedded within tumor nodules, indicating that pre-existing blood flow entered tumors via residual portal tracts. These results indicate the presence of vessel co-option as a blood supply mechanism in iCCAs with replacing HGP, particularly CLC subtypes [18, 23, 32]. In general, proangiogenic factors induce sprouting from pre-existing capillaries in the process of tumorigenesis, and this process is targeted for anticancer treatments.[48] In biliary tract cancer, although hypervascular tumors are not rare, antiangiogenic therapies have not significantly improved patient prognosis, and the presence of vessel co-option adopted by tumor cells might explain this limitation [23, 27, 48, 49].

IHC staining for endothelial antibodies is a potential method to distinguish between neo-angiogenesis and vessel co-option, although no specific targetable marker for endothelial cells of co-opted vessels has been identified [23, 28, 32, 50]. ELTD1 is upregulated in tumor-associated endothelial cells of renal and colorectal cancers, with a high level correlating with a better prognosis, which serves as a predictor [33, 34, 51, 52]. In the present study, ELTD1 was expressed in tumor-related angiogenic vessels and co-opted vessels. ELTD1-positive MVD at the invasive margin in replacing HGP was significantly higher than those in desmoplastic and pushing HGPs (*p* < 0.01) (Fig. 2c). Moreover, ELTD1-positive MVD at the invasive margins in DPM and CLC subtypes was higher than those in the SBD and LBD types (*p* < 0.01) (Fig. 2b). We also observed that larger tumor sizes correlated with lower ELTD1-positive MVD. These findings indicate that the histological types and HGPs are associated with ELTD1-positive MVD, including vessel co-option. Interestingly, in cSBD-CLC cases, ELTD1-positive MVD at the invasive margin (CLC-rich area) was significantly higher than that at the tumor center (SBD-rich area) (Fig. 4b), indicating that the CLC component, which is located at the invasive area and shows replacing HGP, primarily utilizes vessel co-option for blood supply [11]. In contrast, REDD1 expression was limited in tumor-related angiogenic vessels. REDD-1 is generally upregulated under hypoxic conditions and associated with the promotion of angiogenesis and poor prognosis in various cancers [35, 53–57]. The present study revealed that the high REDD1-positive MVD level at the tumor center in iCCA suggests the presence of a large hypoxic core, irrespective of iCCA (sub)types and HGPs. This hypoxia-induced angiogenesis differs from the mechanism of vessel co-option and also characterizes the histogenesis of iCCA.

The present study revealed that both TAGLN and α-SMA were useful for detecting arteries, but TAGLN expression is more limited in arteries. In the DPM subtype, total AVD was high at both the invasive margin and tumor center, which also tended to be higher than those in the background liver. In other iCCA (sub)types, however, total AVD shows a slightly opposite trend, indicating that all iCCA (sub)types except the DPM subtype exhibit arterial blood-poor tumor (Fig. 3a and b, and Supplemental Table 1). Regarding arterial densities among HGPs, iCCAs with different HGPs differed significantly in AVD at both the invasive margin and tumor center with the lowest number in pushing HGP tumors. Despite being comparable in total AVD, desmoplastic HGP tumors had a higher proportion of unpaired AVD/paired AVD at both the invasive margin and tumor center compared with those with replacing HGP. This indicates that the blood supply via angiogenesis is more prominent in desmoplastic HGP, while via vessel co-option it is more prominent in replacing HGP iCCAs. Radiologically, most iCCA nodules are visualized as hypovascular, but some display hypervascularity [26, 58–60]. These hypervascular iCCA nodules show low-grade malignancy from the aspect of the patient’s prognosis and histopathologically including DPM, CLC, or cSBD–CLC subtypes [11, 58–60]. In this study, the DPM subtype constantly demonstrated high MVD and unpaired AVD levels, but also prominent replacing HGPs, suggesting that the hypervascular tumor employs both mechanisms of neo-angiogenesis and vessel co-option [11].

Tumors establish a comprehensive vascular system through blood and lymphatic vessels to promote cell growth and metabolism [61]. In addition to blood vessels, tumor-associated lymphangiogenesis plays a crucial role in a patient’s prognosis as an independent prognostic factor [62]. In this study, high LVD was frequently found in DPM. Although patients with higher LVD generally have worse survival rates in cholangiocarcinoma because of increases in the risk of lymphatic invasion, we speculate that the replacing HGP component of the DPM subtypes results in a mode of co-opted mature lymphatic vessels accompanying blood vessel co-option [62, 63]. In fact, the LVD of replacing HGP was significantly higher than those of desmoplastic and pushing HGPs. An effective immune response via these lymphatic vessels may contribute to the differences in OS of different HGPs and iCCA (sub)types.

Tumor-infiltrating lymphocytes (TILs) and tumor-associated neutrophils (TANs) are the main components of the tumor immune microenvironment [4]. An increased number of CD8 + T cells is associated with a better prognosis, but the presence of CD8 + T cells alone cannot explain the influence of OS on iCCA [58, 64, 65]. Our results revealed that the density of CD8 + T cells at the invasive margin in the DPM subtype and replacing HGPs was higher than that in the background liver. We also noticed lower levels of CD8 + T cells in the hypoxic tumor center, irrespective of iCCA (sub)types and HGPs, indicating that angiogenesis under hypoxic conditions may prevent T cell infiltration within the tumor. In addition to CD8 + T cells, several previous studies revealed that TANs are associated with proangiogenic and immunosuppressive features and predict poor prognosis in iCCA patients [11, 66–68]. In this study, at the invasive margin and tumor center, the number of CD66b + cells of DPM subtype differed from other iCCA (sub)type and showed as low as similar levels at background liver (Fig. 4g, h, and i and Supplemental Table 1), supporting a comparable better prognosis of DPM subtype. To summarize from the perspective of inflammatory cells, iCCA with DPM had a higher CD8 + T cell infiltration and a lower level of CD66b + tumor-associated neutrophils, which explains the better prognosis of this subtype [45].

All these results underscore that HGPs play a pivotal role in the progression of iCCA through vascular mechanisms. The predominant replacing HGP of CLC and DPM subtypes absorbs pre-existing sinusoids (vessel co-option), embeds original portal tracts, enhances tumor hypervascularity, and promotes lymphocytic infiltration. These processes correlate with indicators of a favorable prognosis in iCCA. Here also there are certain limitations. We could not obtain sufficient clinical data, particularly, each patient’s prognosis after the operation, making our correlation between the HGPs, histological (sub)types, and clinical outcomes not totally convincing. Besides, even though a large number of iCCA samples were analyzed, the number of samples in each (sub)type was still limited. Now, it is unclear why the CLC subtype would exploit vessel co-option more readily than other (sub)types, rather than angiogenesis. Henceforth, prospective studies are needed to clarify more drivers’ factors of vessel co-option.

In conclusion, we describe that vessel co-option is a common event in the tumoral blood system in iCCA, especially CLC and DPM subtypes. HGPs could be used to distinguish between angiogenic and non-angiogenic tumors, with considerable prognostic implications. We also provided ELTD1 and REDD1, as combined endothelial markers to detect tumor co-opted and angiogenic vessels. ELTD1 may play an important role in vessel co-option. Hence, understanding the impact of vessel co-option in iCCA may provide new avenues for therapies targeting both angiogenesis and vessel co-option, potentially advancing the treatment of this malignancy.

### Supplementary Information

Below is the link to the electronic supplementary material.

Supplementary file1 (DOCX 43 KB)

Supplementary file2 (PDF 9638 KB)

## Data Availability

The data that support the findings of this study are available on request from the corresponding author. The data are not publicly available due to them containing information that could compromise the privacy of research participants. All data relevant to the study are anonymized, and the risk of identification is minimal. Access to these data will be granted to researchers who provide a methodologically sound proposal and whose proposed use of the data has been approved by an Institutional Review Board (IRB).
