# Supplementary material for: Histopathological growth pattern and vessel co-option in intrahepatic cholangiocarcinoma
Source: Med Mol Morphol. 2024 Jul 3;57(3):200–17. doi: 10.1007/s00795-024-00392-1 (PMC11343874; doi:10.1007/s00795-024-00392-1)
Supplement: Supplementary file 1 — Supplementary file1 (DOCX 43 KB) [file 795_2024_392_MOESM1_ESM.docx]

**Histopathological growth pattern and vessel co-option in intrahepatic cholangiocarcinoma**

Zihan Li, M.D.,^1)^ Hiep Nguyen Canh, M.D., Ph.D,^1)^ Kenta Takahashi, Ph.D,^1)^ Dong Le Thanh, M.D.,^1)^ Quynh Nguyen Thi, M.D.,^1)^ Rui Yang, M.D.,^1)^ Kaori Yoshimura, M.D., Ph.D,^1)^ Yasunori Sato, M.D., Ph.D.,^1)^ Khuyen Nguyen Thi, M.D.,^2)^ Hiroki Nakata, M.D., Ph.D.,^3) 4)^ Hiroko Ikeda, M.D., Ph.D.,^5)^ Kazuto Kozaka, M.D., Ph.D.,^6)^ Satoshi Kobayashi, M.D., Ph.D.,^6)^ Shintaro Yagi, M.D., Ph.D.,^7)^ and Kenichi Harada, M.D., Ph.D.,^1)^

1) Department of Human Pathology, Kanazawa University Graduate School of Medicine, Kanazawa, Japan

2) Center of Pathology and Molecular Biology, National Cancer Hospital, Hanoi, Vietnam

3) Department of Clinical Engineering, Faculty of Health Sciences, Komatsu University, Komatsu, Japan

4) Department of Integrative Cancer Therapy and Urology, Kanazawa University Graduate School of Medical Science, Kanazawa, Japan

5) Department of Diagnostic Pathology, Kanazawa University Hospital, Kanazawa, Japan

6) Department of Radiology, Kanazawa University Hospital, Kanazawa, Japan

7) Department of Hepato-Biliary-Pancreatic Surgery and Transplantation, Kanazawa University, Kanazawa, Japan

**Supplemental Table 1-2**

**Supplemental Table 1.** Correlation of MVD, AVD, LVD, TILs and TANs in iCCA by histological groups

**Supplemental Table 2.** Correlation of MVD, AVD, LVD, TILs, and TANs in iCCA by histopathological growth patterns

**Supplemental Fig. 1**

**Supplemental Fig. 1** Immunohistochemical staining of ELTD1, CD34, REDD1, α-SMA, TAGLN, D2-40, CD8, and CD66b in background liver with HGPs types.

Supplemental Fig.2 Associations between iCCA (sub)types, HGPs, clinical data, and survival data

**Supplemental Table 1.** Correlation of MVD, AVD, LVD, TILs and TANs in iCCA by histological groups

| Histological group | | | | | | | |
| --- | --- | --- | --- | --- | --- | --- | --- |
| Characteristic | LBD | SBD | cSBD-CLC | CLC | DPM | P-value | HCC |
|  | 20 cases | 54 cases | 26 cases | 35 cases | 10 cases |  | 39 cases |
| Age (mean SD), y.o. | 60.5 (2.7) | 64.0 (1.5) | 61.0 (1.7) | 64.5 (2.0) | 67.0 (2.8) | 0.502* |  |
| Median | 61.2 | 64.0 | 61.4 | 63.1 | 66.4 |  |  |
| Range | 43-81 | 38-84 | 50-80 | 44-79 | 48-76 |  |  |
| Gender |  |  |  |  |  | 0.406* |  |
| Percentage of male | 55.60% | 73.90% | 64.00% | 73.30% | 50.00% |  |  |
| Tumor size (mean SD), cm | 4.5 (0.5) | 5.0 (0.4) | 5.2 (0.5) | 4.5 (0.4) | 3.0 (0.6) | 0.114* |  |
| Range | 1.8-8.5 | 1.3-12 | 2.3-10 | 0.7-9 | 1-7.3 |  |  |
| ≤ 2 | 2/15 (13.3%) | 4/46 (8.7%) | 0/24 (0.0%) | 3/29 (10.3%) | 3/10 (30.0%) | **0.035**** |  |
| > 2 ~ ≤ 5 | 9/15 (60.0%) | 23/46 (50.0%) | 11/24 (45.8%) | 16/29 (55.2%) | 6/10 (60.0%) |  |  |
| >5 | 4/15 (26.7%) | 19/46 (41.3%) | 13/24 (54.2%) | 10/29 (34.5%) | 1/10 (10.0%) |  |  |
| AJCC 8th edn Stage |  |  |  |  |  | 0.398* |  |
| Stage 1 | 3/15 (20.0%) | 16/45 (35.6%) | 9/24 (37.5%) | 12/29 (41.1%) | 4/10 (40.0%) |  |  |
| Stage 2 | 1/15 (6.7%) | 13/45 (28.9%) | 5/24 (20.8%) | 7/29 (24.1%) | 2/10 (20.0%) |  |  |
| Stage 3 | 11/15 (73.3%) | 14/45 (33.3%) | 7/24 (29.2%) | 8/29 (27.6%) | 4/10 (40.0%) |  |  |
| Stage 4 | 0/15 (0.0%) | 2/45 (4.4%) | 3/24 (12.5%) | 2/29 (6.9%) | 0/10 (0.0%) |  |  |
| Histological growth pattern |  |  |  |  |  |  |  |
| Pushing | 3 (15.0) | 7 (13.0) | 2 (7.7) | 2 (5.7) | 1 (10.0) | **0.003**** |  |
| Desmoplastic | 11 (55.0) | 16 (29.6) | 2 (7.7) | 4 (11.4) | 2 (20.0) |  |  |
| Replacing | 6 (30.0) | 31 (57.4) | 22 (84.6) | 29 (82.9) | 7 (70.0) |  |  |
| Microvessel Density |  |  |  |  |  |  |  |
| ELTD1 |  |  |  |  |  |  |  |
| Background Liver | 44.5 | 42.4 | 35.0 | 41.9 | 34.7 | 0.945* | 61.5 |
| Invasive Margin | 67.5 | 103.6 | 108.8 | 119.7 | 130.4 | **< 0.01*** | 198.2 |
| Tumor Center | 53.3 | 85.4 | 72.7 | 72.3 | 135.0 | **0.016*** | 204.4 |
| CD34 |  |  |  |  |  |  |  |
| Background Liver | 77.0 | 68.5 | 67.8 | 60.7 | 47.4 | 0.663* | 62.6 |
| Invasive Margin | 67.0 | 103.3 | 104.5 | 122.3 | 136.7 | 0.133* | 194.8 |
| Tumor Center | 80.6 | 76.3 | 75.6 | 75.9 | 114.4 | 0.878* | 185.2 |
| REDD1 |  |  |  |  |  |  |  |
| Background Liver | 11.3 | 11.0 | 10.3 | 11.3 | 15.7 | 0.566* | 21.9 |
| Invasive Margin | 13.5 | 16.7 | 19.7 | 28.1 | 41.5 | 0.215* | 64.8 |
| Tumor Center | 21.2 | 34.3 | 35.8 | 36.0 | 59.5 | 0.169* | 68.2 |
| Arterial Vessel Density |  |  |  |  |  |  |  |
| a-SMA |  |  |  |  |  |  |  |
| Background Liver | 23.3 | 15.8 | 22.4 | 14.8 | 18.9 | **0.024*** | 18.9 |
| Invasive Margin |  |  |  |  |  |  |  |
| Total Artery | 12.9 | 16.2 | 16.9 | 17.0 | 27.5 | 0.325* | 18.7 |
| Unpaired Artery | 5.9 (45.7) | 6.7 (41.4) | 6.3 (37.3) | 4.4 (25.9) | 11.7 (42.5) | 0.106* | 9.6 (51.3) |
| Paired Artery | 7.0 (54.3) | 9.5 (58.6) | 10.6 (62.7) | 12.6 (74.1) | 15.8 (57.5) | 0.362* | 9.1 (48.7) |
| Tumor Center |  |  |  |  |  |  |  |
| Total Artery | 12.6 | 14.8 | 16.3 | 16.7 | 30.2 | 0.197* | 23.4 |
| Unpaired Artery | 6.7 (53.2) | 7.2 (48.6) | 7.6 (46.6) | 5.6 (33.5) | 10.6 (35.1) | 0.519* | 16.9 (72.2) |
| Paired Artery | 5.9 (46.8) | 7.6 (51.4) | 8.7 (53.4) | 11.1 (66.5) | 19.6 (64.9) | 0.054* | 6.5 (27.8) |

**Supplemental Table 1.**  (Continued)

| TAGLN |  |  |  |  |  |  |  |
| --- | --- | --- | --- | --- | --- | --- | --- |
| Background Liver | 12.6 | 12.2 | 13.6 | 11.8 | 11.1 | 0.961* | 22.2 |
| Invasive Margin |  |  |  |  |  |  |  |
| Total Artery | 8.8 | 11.9 | 8.0 | 8.8 | 23.4 | 0.186* | 16.7 |
| Unpaired Artery | 3.9 (44.3) | 5.2 (43.7) | 2.5 (31.2) | 2.5 (28.4) | 11.9 (50.9) | **0.039*** | 11.5 (68.9) |
| Paired Artery | 4.9 (55.7) | 6.7 (56.3) | 5.5 (68.8) | 6.3 (71.6) | 11.5 (49.1) | 0.278* | 5.2 (31.1) |
| Tumor Center |  |  |  |  |  |  |  |
| Total Artery | 7.3 | 10.2 | 5.1 | 6.8 | 15.2 | 0.145* | 14.4 |
| Unpaired Artery | 4.6 (63.0) | 4.6 (45.1) | 2.5 (49.0) | 2.2 (32.4) | 8.5 (55.9) | **0.042*** | 11.1 (77.1) |
| Paired Artery | 2.7 (37.0) | 5.6 (54.9) | 2.6 (51.0) | 4.6 (67.6) | 6.7 (44.1) | **0.013*** | 3.3 (22.9) |
| Lymphatic vessel density |  |  |  |  |  |  |  |
| D2-40 |  |  |  |  |  |  |  |
| Background Liver | 12.2 | 18.3 | 28.7 | 21.5 | 17.8 | **0.022*** | 21.1 |
| Invasive Margin | 19.3 | 20.7 | 25.2 | 22.4 | 26.7 | 0.327* | 25.9 |
| Tumor Center | 18.9 | 17.6 | 21.1 | 23.8 | 38.5 | **0.022*** | 23.7 |
| Immune cells density |  |  |  |  |  |  |  |
| CD8 |  |  |  |  |  |  |  |
| Background Liver | 702.2 | 345.9 | 442.2 | 382.8 | 195.8 | **0.043*** |  |
| Invasive Margin | 678.5 | 533.6 | 602.1 | 662.3 | 558.6 | 0.702* |  |
| Tumor Center | 169.1 | 212.7 | 220.0 | 191.2 | 248.5 | 0.974* |  |
| CD66b |  |  |  |  |  |  |  |
| Background Liver | 61.5 | 66.6 | 89.7 | 104.5 | 68.7 | 0.502* |  |
| Invasive Margin | 267.0 | 203.4 | 213.2 | 222.9 | 103.8 | 0.653* |  |
| Tumor Center | 217.6 | 171.7 | 125.6 | 81.4 | 105.1 | 0.408* |  |

* Kruskal-Walli’s test; ** χ test; LBD, large bile duct; SBD, small bile duct; CLC, cholangiolocarcinoma; DPM, ductal plate malformation; cSBD-CLC, combined SBD-CLC

**Supplemental Table 2.** Correlation of MVD, AVD, LVD, TILs, and TANs in iCCA by histopathological growth patterns

| Histological growth pattern n(%) | | | | |
| --- | --- | --- | --- | --- |
| Characteristic | Pushing | Desmoplastic | Replacing | P-value |
|  | 15 cases | 35 cases | 95 cases |  |
| Age (mean SD), y.o. | 63.5 (2.3) | 61.0 (2.2) | 64.5 (1.1) | 0.324* |
| Median | 60.3 | 61.8 | 64.0 |  |
| Range | 46-72 | 41-84 | 38-84 |  |
| Gender |  |  |  | 0.944* |
| percentage of males | 10/14 (71.4%) | 20/30 (66.7%) | 57/85 (67.1%) |  |
| Tumor size (mean SD), cm | 7.3 (0.7) | 3.8 (0.4) | 4.5 (0.2) | **0.002*** |
| Range | 3.7-12.0 | 0.7-8.5 | 1.0-12.0 |  |
| ≤ 2 | 0/13 (0.0%) | 4/26 (15.4%) | 8/85 (9.4%) | **0.006**** |
| > 2 ~ ≤ 5 | 3/13 (23.1%) | 15/26 (57.7%) | 47/85 (55.3%) |  |
| >5 | 10/13 (76.9%) | 7/26 (26.9%) | 30/85 (35.3%) |  |
| AJCC 8th edn Stage |  |  |  | **0.036**** |
| Stage 1 | 2/13 (15.4%) | 9/26 (34.6%) | 33/85 (38.8%) |  |
| Stage 2 | 1/13 (7.7%) | 5/26 (19.2%) | 22/85 (25.9%) |  |
| Stage 3 | 8/13 (61.5%) | 12/26 (46.2%) | 25/85 (29.4%) |  |
| Stage 4 | 2/13 (15.4%) | 0/26 (0.0%) | 5/85 (5.9%) |  |
| Microvessel Density |  |  |  |  |
| ELTD1 |  |  |  |  |
| Background Liver | 29.3 | 61.2 | 44.0 | **< 0.01*** |
| Invasive Margin | 61.2 | 91.1 | 130.0 | **< 0.01*** |
| Tumor Center | 36.4 | 107.8 | 77.3 | **< 0.01*** |
| CD34 |  |  |  |  |
| Background Liver | 61.1 | 33.3 | 68.1 | 0.148* |
| Invasive Margin | 69.3 | 100.0 | 127.8 | **0.020*** |
| Tumor Center | 54.3 | 91.5 | 84.6 | 0.118* |
| REDD1 |  |  |  |  |
| Background Liver | 10.5 | 16.0 | 12.1 | 0.111* |
| Invasive Margin | 11.6 | 18.3 | 27.3 | 0.064* |
| Tumor Center | 24.8 | 40.4 | 32.8 | 0.342* |
| Arterial Vessel Density |  |  |  |  |
| a-SMA |  |  |  |  |
| Background Liver | 17.8 | 22.6 | 20.0 | 0.332* |
| Invasive Margin |  |  |  |  |
| Unpaired arteries | 3.3 (33.0) | 7.0 (42.2) | 6.7 (34.0) | **0.007*** |
| Paired arteries | 6.7 (67.0) | 9.6 (57.8) | 13.0 (66.0) | **< 0.01*** |
| Total arteries | 10.0 | 16.6 | 19.7 | **< 0.01*** |
| Tumor Center |  |  |  |  |
| Unpaired arteries | 3.0 (40.5) | 8.5 (52.1) | 7.0 (37.8) | **0.024*** |
| Paired arteries | 4.4 (59.5) | 7.8 (47.9) | 11.5 (62.2) | **< 0.01*** |
| Total arteries | 7.4 | 16.3 | 18.5 | **< 0.01*** |
| TAGLN |  |  |  |  |
| Background Liver | 10.5 | 15.9 | 12.7 | 0.171* |
| Invasive Margin |  |  |  |  |
| Unpaired arteries | 2.2 (39.3) | 6.0 (46.2) | 4.9 (40.8) | **0.020*** |
| Paired arteries | 3.4 (60.7) | 7.0 (53.8) | 7.1 (59.2) | **< 0.01*** |
| Total arteries | 5.6 | 13.0 | 12.0 | **< 0.01*** |
| Tumor Center |  |  |  |  |
| Unpaired arteries | 2.2 (59.5) | 4.9 (55.7) | 4.9 (43.8) | **0.014*** |
| Paired arteries | 1.5 (40.5) | 3.9 (44.3) | 6.3 (56.2) | **< 0.01*** |
| Total arteries | 3.7 | 8.8 | 11.2 | **< 0.01*** |

**Supplemental Table 2.** (Continued)

| Lymphatic vessel density |  |  |  |  |
| --- | --- | --- | --- | --- |
| D2-40 |  |  |  |  |
| Background Liver | 17.4 | 18.9 | 23.2 | 0.296* |
| Invasive Margin | 16.9 | 20.6 | 25.6 | **0.025*** |
| Tumor Center | 15.4 | 15.9 | 27.0 | **< 0.01*** |
| Immune cells density |  |  |  |  |
| CD8 |  |  |  |  |
| Background Liver | 576.0 | 337.7 | 396.6 | 0.368* |
| Invasive Margin | 508.6 | 609.9 | 673.2 | 0.400* |
| Tumor Center | 217.8 | 200.5 | 199.9 | 0.956* |
| CD66b |  |  |  |  |
| Background Liver | 74.3 | 85.8 | 81.0 | 0.900* |
| Invasive Margin | 130.0 | 236.7 | 230.6 | 0.599* |
| Tumor Center | 133.2 | 196.0 | 125.5 | 0.494* |

* Kruskal-Walli’s test; ** χ test

**Supplemental Figure legend**

**Supplemental Fig. 1** **Immunohistochemical staining of ELTD1, CD34, REDD1, α-SMA, TAGLN, D2-40, CD8, and CD66b in background liver with HGPs types.**

The representative images of IHC staining for background liver by ELTD1 (a, x200), CD34 (b, x200), REDD1 (c, x200), α-SMA (d, x100), TAGLN (e, x100), D2-40 (f, x200), CD8 (g, x200), and CD66b (h, x200) in background liver with HGPs types.

(IHC immunohistochemical staining, HGPs histopathological growth patterns)

**Supplemental Fig. 2 Associations between iCCA (sub)types, HGPs, clinical data, and survival data**

a. Scatter plot illustrating the correlation between tumor size and proportion of HGP, with linear regression analysis. b. Scatter plot depicting the relationship between AJCC stage proportions and HGP, with linear regression analysis. c. Scatter plot showing the correlation between MVD/LVD values at invasive margin and tumor size, with linear regression analysis. d. Scatter plot presenting the relationship between MVD/LVD values at tumor center and tumor size, with linear regression analysis. e. Overall survival analysis based on HGP typing in iCCA. f. Overall survival analysis by histologic (sub)types in iCCA. g. Disease-free survival analysis according to HGP typing in iCCA. h. Disease-free survival analysis based on histologic (sub)types in iCCA.

(Survival data after the operation were available for 63 patients, log-rank test was used to compared groups.)
