# Supplementary figures and images for: Histopathological growth pattern and vessel co-option in intrahepatic cholangiocarcinoma
Source: Med Mol Morphol. 2024 Jul 3;57(3):200–17. doi: 10.1007/s00795-024-00392-1 (PMC11343874; doi:10.1007/s00795-024-00392-1)

Supplement Fig. 1

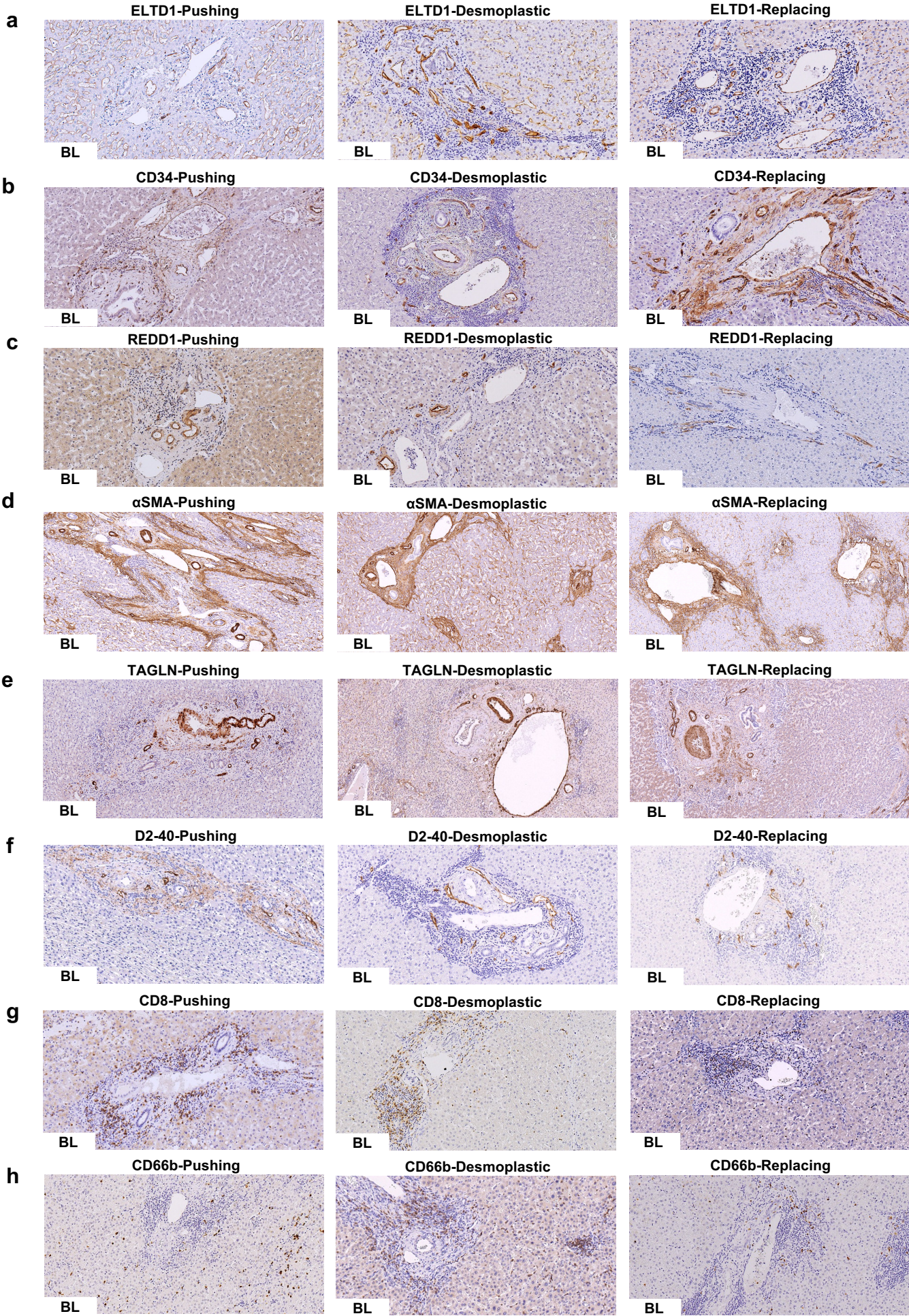

Supplement Fig. 2

a

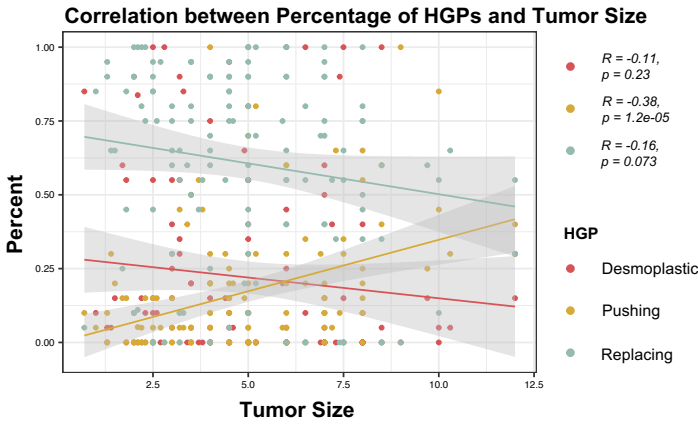

b

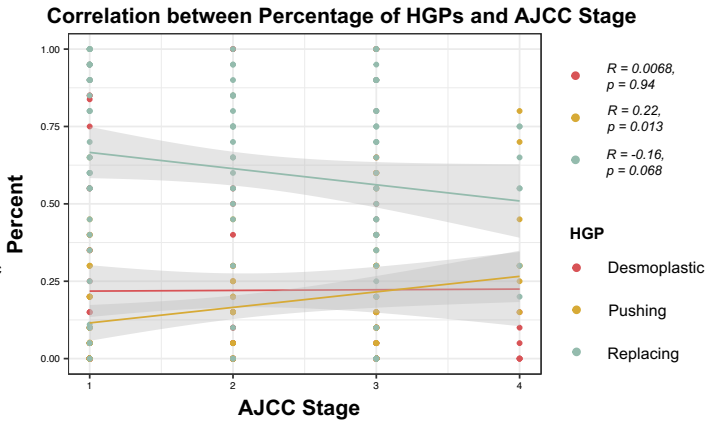

c

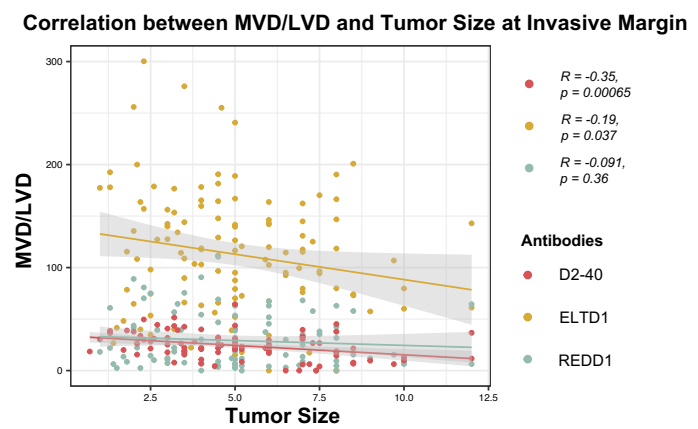

d

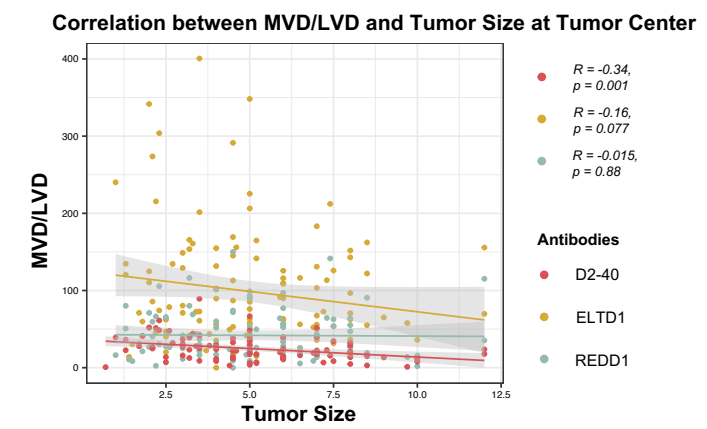

e

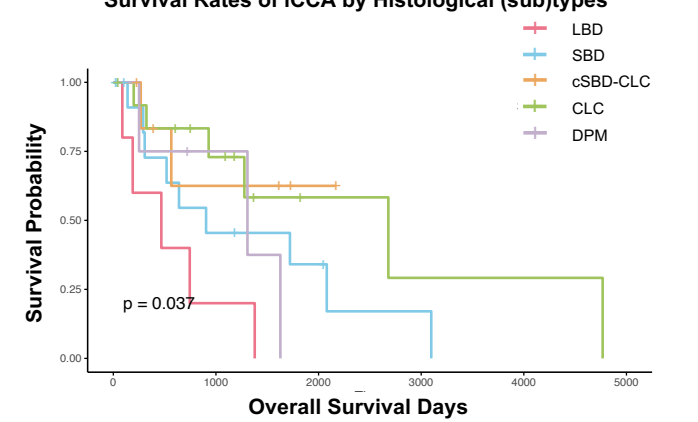

f

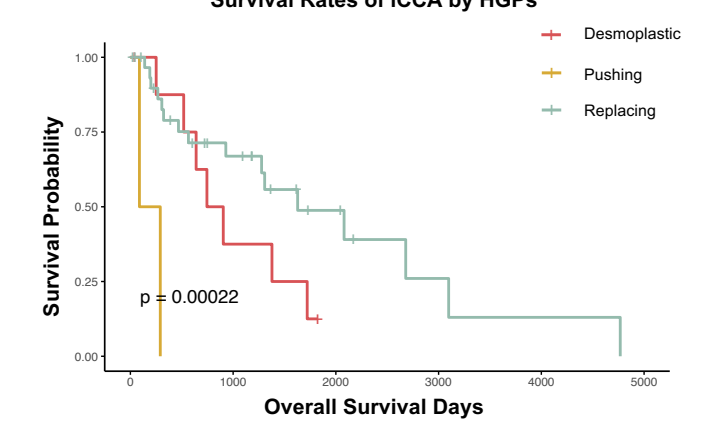

g

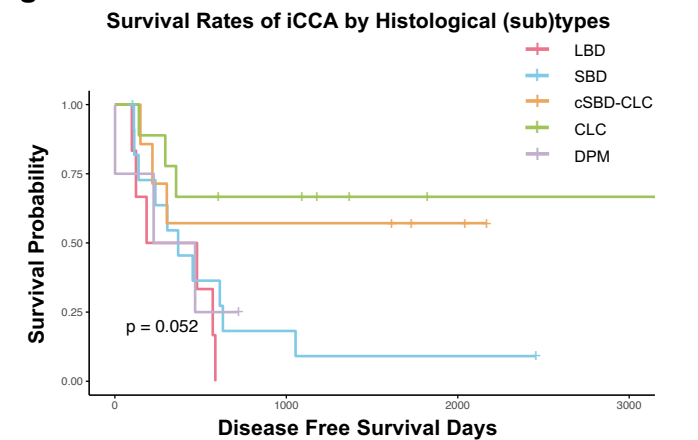

h

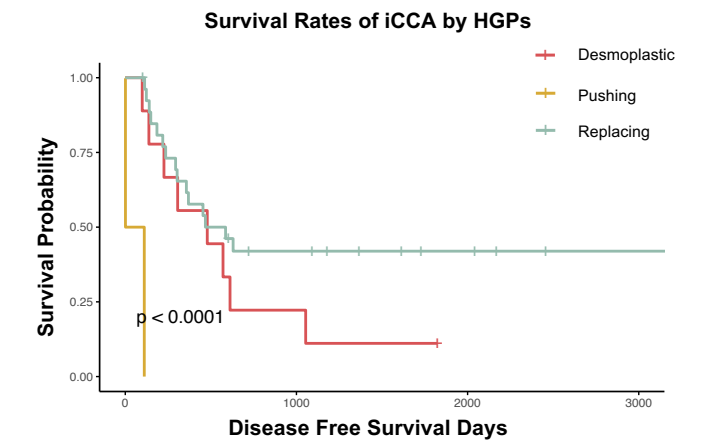

Supplement: Supplementary file 2 — Supplementary file2 (PDF 9638 KB) [file 795_2024_392_MOESM2_ESM.pdf]
